# The Air We Breathe

**Published:** 1875-11

**Authors:** 


### THE AIR WE BREATHE.

The atmosphere is composed of one part oxygen and four parts nitrogen. The former supports life, the latter extinguishes it. The more oxygen there is, the livelier, the healthier, and the more joyful are we; the more nitrogen, the more sleepy, and stupid, and dull, do we become. But if all the air were oxygen, the first lighted match would wrap the world in instant flame; if all were nitrogen, the next instant there would not be upon the populated globe a single living creature.

When oxygen was discovered by Priestley, nearly a century ago, there was a universal jubilation among doctors and chemists. The argument was plausible, and seemed perfectly convincing—"If oxygen is the life and health of the atmosphere, as we have found out how to make oxygen, we have only to increase the quantity in the air we breathe, in order to wake up new life, to give health to the diseased, and youth to the aged." But, on trial, it was found that it made a man a maniac or a fool, and, if continued, a corpse! Various other experiments have been made to improve upon the handiwork of the all-wise Maker of the universe, but they have been successive failures; and thinking men have long since come to the conclusion, that, as there can be no improvement upon the cold water of the first creation, in slaking thirst, so there can no addition be made to pure air, which will better answer its life-sustaining purposes. And as there is not, in all nature, a still, warm atmosphere, that does not instantly begin to generate decay, corruption, and death, so there is no chamber of the sick, graduated to a degree and with air unchanged, that will not hasten the end desired to be averted. Nor is there an atom in nature which can add to the health and life-giving influence of the pure air of heaven; for if it displaces the oxygen, in the same proportion does it diminish its life; and if it displaces the nitrogen, just to the same extent does it lessen the conservative power of nature, and kindle a fever which is to burn up the body.

